# Evaluation of Anterior Decompression Surgical Outcomes of Proximal‐Type Cervical Spondylotic Amyotrophy: A Retrospective Study

**DOI:** 10.1111/os.12654

**Published:** 2020-04-15

**Authors:** Chang‐bo Lu, Zhen‐sheng Ma, Jin‐bo Hu, Xiao‐jiang Yang, Wei Wei, Yang Zhang, Wei Lei

**Affiliations:** ^1^ Department of Orthopaedic Surgery Xijing Hospital, Air Force Medical University Xi'an China

**Keywords:** Cervical spondylotic amyotrophy, Anterior decompression, Surgical outcomes

## Abstract

**Objective:**

To investigate the efficiency of anterior decompression on the proximal‐type cervical spondylotic amyotrophy patients.

**Methods:**

This was a retrospective analysis. From January 2014 to November 2017, 21 patients with proximal‐type cervical spondylotic amyotrophy (CSA) underwent anterior decompression. There were 15 males and 6 females, aged 35–73 years with an average of 51.62 years. All the patients underwent surgery of anterior decompression (ACDF or ACCF). Among them, 12 patients underwent C_4/5_ single level ACDF, eight patients underwent C_4/5_ and C_5/6_ double level ACDF, and one patient underwent C5 anterior cervical corpectomy decompression and fusion surgery. Preoperative and postoperative clinical and radiologic parameters were assessed. The clinical examinations were reviewed, including muscle strength, neck disability index (NDI) score, cervical Japanese Orthopaedic Association (JOA) score, and improvement rate of manual muscle test (MMT) at the last follow‐up. Preoperative spinal cord or nerve impingement was assessed by magnetic resonance imaging (MRI) or computed tomography (CT) myelography. Postoperative lateral X‐ray radiographs were performed every 3 months after the surgery.

**Results:**

Severe preoperative muscle atrophy of the deltoid or biceps muscles occurred in 21 patients included in the study. All of them involve impingements of the ventral nerve root and/or the anterior horn according to MRI and CT myelography. The preoperative duration of symptoms averaged 8.4 months. The average follow‐up for all patients was 13.2 months. At the final follow‐up, all patients showed statistically significant improvements in muscle strength and NDI scores (*P* < 0.05, *P* < 0.05). For the deltoid muscles force and C‐JOA scores, the average improvement rates were 66.49% ± 10.04% and 62.23% ± 9.23%, respectively. With respect to MMT, 12 proximal‐type patients were graded excellent, six were good, and three were fair, and the overall improvement rate was 85.7%.

**Conclusions:**

For proximal‐type CSA patients with cervical radiculopathy, earlier anterior decompression surgery can achieve satisfactory results by significantly improving a patient's muscle strength and relieving compression symptoms.

## Introduction

Cervical spondylotic amyotrophy (CSA) is an uncommon clinical syndrome which is characterized by muscle atrophy in the upper extremities with no or insignificant sensation deficits. Although such a clinical presentation has been described by Brain *et al*. in 1952 as muscle atrophy of the upper limbs without sensory disturbance^1^, the first case of cervical spondylotic amyotrophy as a clinical syndrome of “dissociated motor loss in the upper extremities with cervical spondylosis” was reported by Keegan in 1965^2^, ^3^. Cervical spondylotic amyotrophy is classified into two subtypes according to the most predominantly affected muscle groups: proximal amyotrophy (deltoid and biceps) and distal amyotrophy (triceps, forearm, and hand muscles). Muscular atrophy of the proximal‐type patients is mainly localized in the C_5_ and C_6_ myotomes^2^, ^4^, ^5^, while for patients of the distal‐type, the responsible lesion involves the anterior horn at C_7_‐T_1_ myotomes. Clinically, most cervical spondylotic amyotrophy patients are involved in unilateral disorder whereas few others are bilateral symmetric disorder^6^.

Cervical spondylotic amyotrophy often follows a self‐limited course, which means that the manifestations usually keep steady for years after an initial progressive course. Typically, sensory loss or pyramidal signs are absent or insignificant in cervical spondylotic amyotrophy patients. Thus, diagnosis of cervical spondylotic amyotrophy mainly depends on a combination of radiographic examination, clinical manifestation, disease course, and electrophysiological findings. In the treatment of CSA, conservative intervention was once suggested at the onset of neurological symptom and seems to hamper disease progression for some proximal‐type cervical spondylotic amyotrophy. Whereas in some other studies, surgical intervention is recommended if conservative treatment has not been successful^7^, ^8^. In exploring the prognostic factors for cervical spondylotic amyotrophy, several recent studies have found positive correlations between the long duration of symptoms and the poor surgical outcome regardless of the proximal‐type or the distal‐type^9^, ^10^, ^11^. Thus early surgical intervention is recommended once diagnosed in a more recent study^12^.

**Figure 1:**
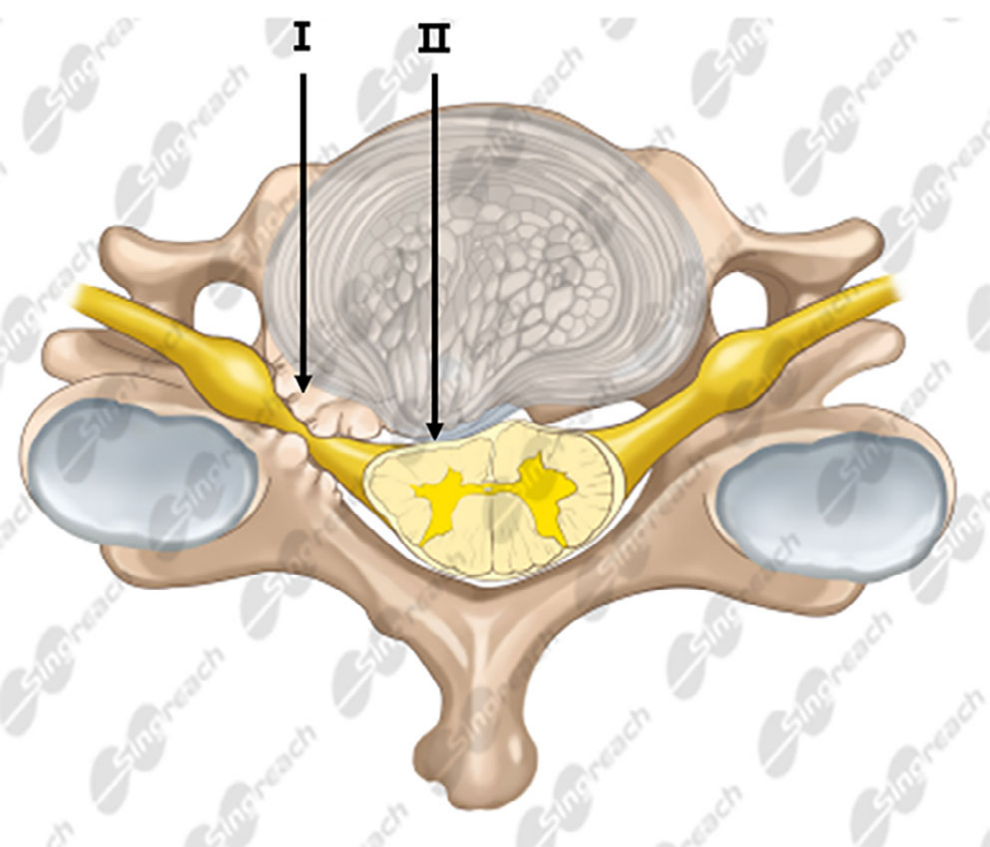
Possible pathogenesis of cervical spondylotic amyotrophy. I: impingement against ventral root; II: impingement against anterior horn.

Indeed, the underlying pathogenesis of cervical spondylotic amyotrophy is still not fully understood. The focus lies in whether cervical spondylotic amyotrophy is the result of selective damage to the ventral root or to anterior horn (Fig. 1), two essentially different pathogenesis for CSA^13^. Also, clinical efficiency of improvement in muscle strength of upper extremity varies across patients who underwent surgical treatments. Although predictive factors involving the prognosis of anterior decompression was investigated by some studies^14^, ^15^, yet few of these studies distinguish damage to the ventral nerve root or the anterior horn. Indeed, the two types of damage belong to different pathophysiologies^16^. In the clinical practice, patients diagnosed with cervical spondylotic amyotrophy often manifest either the myelopathy or radiculopathy^2^, ^17^, but it is difficult to distinguish underlying pathophysiologies from magnetic resonance imaging (MRI) for a large proportion of CSA patients. Thus, awareness is still lacking about clinical features of either type of cervical spondylotic amyotrophy, and associations between surgical procedures and outcomes have not been fully understood.

In this study, we made an effort to review proximal‐type cervical spondylotic amyotrophy patients admitted to our hospital for treatment and identified the cases especially caused by radiculopathy on the basis of MRI findings. The purposes of this study were, firstly, to summarize current understanding and the clinical features of CSA, and secondly, to clarify the efficiency of anterior decompression approach for the proximal‐type cervical spondylotic amyotrophy patients with radiculopathy.

## Material and Methods

### *Patients*

Written informed consent was obtained from each patient and the study was approved by the Ethics Committee of Xijing Hospital, Xi'an, China. The inclusion criteria followed the PICOS principle: (i) patients diagnosed with CSA treated in our center; (ii) the CSA was treated by surgery of anterior decompression (ACDF or ACCF); (iii) preoperative and the postoperative comparisons were made with items including imaging data, muscle strength, neck disability index (NDI) score, cervical Japanese Orthopedic Association (JOA) score, and improvement rate of manual muscle test (MMT); (iv) improvements in the results of long‐term outcome between pre‐operation and post‐operation should be expected; and (v) the study design was a retrospective study. The exclusion criteria for this study are as follows: (i) brachial plexus and peripheral nerve injury (carpal tunnel syndrome, cubital tunnel syndrome, biceps tendonitis, thoracic outlet Syndrome, etc.); (ii) cervical flexion myelopathy multifocal motor neuropathy; and (iii) amyotrophic lateral sclerosis. Patients diagnosed as CSA must meet the following criteria: (i) the presence of unilateral muscle atrophy or impairment of the shoulder girdle muscles; (ii) mild or no sensory disturbance in the upper extremity; (iii) MRI or computed tomography (CT) indicating nerve root compression and/or spinal cord compression; and (iv) no gait disturbance.

From January 2014 to November 2017, 21 cases subjects in our department who had proximal‐type CSA were enrolled in our study. All patients complained of radicular pain or numbness in unilateral scapular area, shoulder or upper arm which lasted for 8.8 weeks (2–17 weeks) on average.

We collected data on patients' sex, age, surgical approaches, preoperative manual muscle test (MMT) results, C‐JOA scores, NDI score, duration of symptoms, levels of spinal canal stenosis, etiological diagnosis, and presence of high‐intensity zones on T2‐weighted MRI.

### *Surgical Procedure*

All patients underwent general anesthesia in supine position using tracheal intubation. Surgeries began with incision of the right anterior cervical skin, then subcutis and muscular layer were bluntly dissected. The front of the vertebral body was exposed along the intermuscular space. Localized by fluoroscopy examination, the target intervertebral space was distracted and ACDF or ACCF surgery was performed according to the surgical plan. During the surgery, a complete decompression was performed to the Luschka joint especially in the affected side. Additional osteophytectomy was then performed to enlarge the intervertebral foramen, and the spinal canal or nerve root canal was carefully explored to see whether there was broken nucleus pulposus tissue. Finally, cages or titanium mash filled with autologous bone grains were placed into the intervertebral space and anterior titanium plate fixation was performed to obtain firm interfusion.

Among the 21 cases included in our analysis, 13 patients underwent C4/5 single‐segment ACDF surgery, seven patients underwent C4/5 and C5/6 dual‐segment ACDF surgery, and one underwent C5 vertebral ACCF surgery.

### *Assessment of Surgical Outcome*

For these patients, we collected the basic information including operative procedure, operation time, blood loss during the cervical operation, and postoperative hospital stay. All imaging studies include plain radiographs (neutral, flexion, and extension views), CT and MRI examinations were performed to identify the presence of impingement of the ventral nerve root and corresponding intervertebral levels in each patient. To evaluate the effect of surgical treatment, we measured items including manual muscle testing (MMT), neck disability index (NDI), and cervical Japanese Orthopedic Association (C‐JOA) score.

#### *Manual Muscle Testing (MMT)*

Manual muscle testing (MMT) was adopted to quantify the muscle strength. MMT improvement rate = (last follow‐up muscle strength – preoperative muscle strength) / (5 – preoperative muscle strength). Improvements in muscle power of the most severely atrophic muscle were classified into four grades: excellent (more than two grades of recovery on manual muscle testing), good (one grade of improvement on manual muscle testing), fair (no improvement on manual muscle testing), and poor (worsening on manual muscle testing).

#### *Neck Disability Index (NDI)*

The Neck Disability Index (NDI) is a 10‐item questionnaire that assesses disability associated with neck pain and whiplash (NDI‐Vernon and Mior 1991). A score of less than 0–4 indicates no disability, 5–14 mild disability, 15–24 moderate disability, 25–34 severe disability, and scores greater than 35 complete disability.

#### *Cervical Japanese Orthopaedic Association Score of Cervical Spine (C‐JOA)*

C‐JOA is one of the most frequently used clinical outcome measures to quantify functional status in patients with cervical myelopathy. A score of 16–17 indicates normal function (best possible outcome), a score of 12–15 is grade 1, a score of 8–11 is grade 2, and a score of 0–7 is the most severe deficits. The recovery rate for the C‐JOA score = [(postoperative score – preoperative score)/(17‐preoperative score)]×100%.

### *Statistical Analysis*

The statistical analysis of the clinical data was performed with software SPSS version 24.0 (SPSS, Inc., Chicago, IL). Measured data were expressed as mean ± standard deviation (x ± s). Variations of postoperative follow‐up were analyzed by Wilcoxon signed‐rank test, of which *P* < 0.05 was considered a significant difference.

## Results

All the proximal‐type CSA patients in our study underwent anterior cervical discectomy and fusion (ACDF) or anterior cervical corpectomy and fusion (ACCF). Surgeries were performed by the same surgeon to ensure consistency in variables. After an average follow‐up of 13.2 months (10–32 months), four individuals were lost to follow‐up. Eventually, 21 subjects (15 males and six females) aged 35–73 years were included in this study, with an average age of 52.9 years.

### *The General Surgical Outcomes*

**Figure 2:**
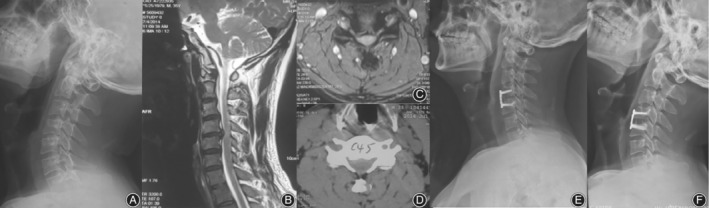
A 35‐year‐old male patient with proximal‐type CSA at the C_4/5_ level. (A) The pre‐operative X‐rays of the cervical spine showing narrowed intervertebral space of the C_4/5_. (B) Sagittal T2‐weighted magnetic resonance image showing cord compression at the C_4_‐C_5_ space; (C) Axial T2‐weighted magnetic resonance image at the C_4_‐C_5_ space showing impingement against the right ventral nerve root and anterior horn, with no abnormal signal intensity in the spinal cord. (D) CT of the cervical spine show that the center of the C_4/5_ disc protrudes to the right, the outlet of the right nerve root is narrowed. (E) postoperative lateral X‐ray of the cervical spine showing that the internal fixation is firm. (F) Follow‐up at 3 months, X‐ray of the cervical spine showing that the internal fixation is firm and the bony fusion is formed.

**Figure 3:**
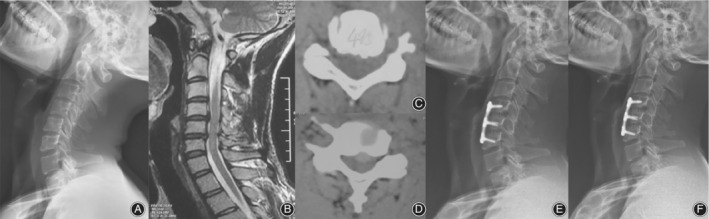
A 41‐year‐old male patient with proximal‐type CSA at the C_4/5_ and C_5/6_ levels. (A) The pre‐operative X‐rays of the cervical spine showing narrowed intervertebral space of the C_4/5_ and C_5/6_ levels. (B) Sagittal T2‐weighted magnetic resonance image showing cord compression at the C_4_‐C_6_ space. (C) CT image at the C_4_‐C_5_ space showing impingement against C_4/5_ intervertebral disc on the left side. (An intraoperative exploration confirmed that multiple nucleus pulposus broke into the spinal canal and the nerve root outlet). (D) CT image at the C_5_‐C_6_ space showing that the central C_5/6_ disc protruded to the left and the outlet of the left nerve root narrowed. (E) postoperative lateral X‐ray of the cervical spine showing that the internal fixation is in good position. (F) Follow‐up at 6 months, X‐ray of the cervical spine showing that the internal fixation is firm and solid arthrodeses is formed.

**Figure 4:**
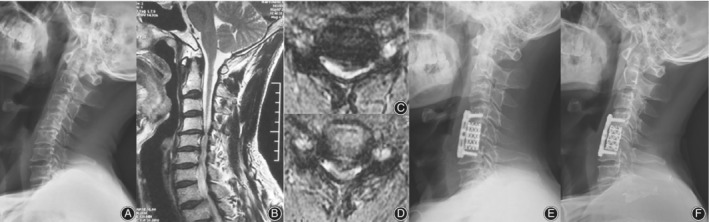
A 48‐year‐old male patient with proximal‐type CSA at the C_4/5_ and C_5/6_ levels. (A) The pre‐operative X‐rays of the cervical spine showing cervical hyperplasia bone hyperplasia, narrowed C_4/5_ and C_5/6_ intervertebral space. (B) Sagittal T2‐weighted magnetic resonance image showing cord compression at the C_4_‐C_6_ space. (C) Axial T2‐weighted magnetic resonance image at the C_4_‐C_5_ space showing both left ventral nerve root nerve root and spinal cord were compressed at the C_4‐5_ intervertebral level. (D) Axial T2‐ weighted magnetic resonance image at the C_5_‐C_6_ space showing narrowed segmental spinal canal and impingement against the left anterior horn nerve roots (white arrows). (E) Postoperative lateral X‐ray of the cervical spine showing that the internal fixation and the titanium cage were in appropriate position. (F) Follow‐up at 3 months, X‐ray of the cervical spine showing that the internal fixation is firm and maintained in appropriate position.

The patients' characteristics and operation approaches are shown in Table 1. Of the 21 patients diagnosed as CSA, the average operation time was 136.7 min, the average intraoperative blood loss was 111.9 mL, and the postoperative hospital stay was 4.63 days on average. For 13 patients who underwent C4/5 ACDF, prolapse intervertebral disc can be found in the spinal canal, resulting in foramen stenosis or oppression of ipsilateral nerve root (Fig. 2). For seven patients who underwent C_4/__5_, C_5/6_ two‐stage ACDF patients, different degrees of ipsilateral intervertebral disc protrusion in intervertebral space and nerve root compression can be found (Fig. 3). It should also be noted that in one case, aside from a huge deviation of the contralateral disc herniation, spinal canal stenosis was found during the surgical procedure (Fig. 4), thus C_5_ ACCF was performed to achieve a thorough decompression.

**Table 1:** Clinical and imaging data

Number	gender/age (years)	Lesion site	etiological diagnosis	Site of compression in MRI	duration of symptoms(months)	surgical approaches	operating time (min)	hemorrhage volume (mL)
1	Male/41	C_4/5_ C_5/6_	CDH	NCR	8	C_4‐6_ ACDF	140	150
2	Male/48	C_4/5_	CDH	NCR	2	C_4/5_ ACDF	135	70
3	Female/59	C_4/5_ C_5/6_	CDH	SCC	6	C_4‐6_ ACDF	156	100
4	Male/54	C_4/5_	CDH	NCR	10	C_4/5_ ACDF	121	180
5	Male/60	C_4/5_C_5/6_	CDH	NCR	16	C_4‐__6_ ACDF	152	130
6	Male/35	C_4/5_	CDH	NCR	4	C_4/5_ ACDF	126	70
7	Female/62	C_4/5_ C_5/6_	CDH	NCR and SCC	12	C_4‐__6_ ACDF	138	120
8	Male/49	C_4/5_	CDH	NCR	15	C_4/5_ ACDF	128	100
9	Male/56	C_4/5_	CDH	SCC	10	C_4/5_ ACDF	145	90
10	Male/73	C_4/5_	CDH	NCR	9	C_4/5_ ACDF	150	170
11	Female/48	C_4/5_	CDH	NCR	7	C_4/5_ ACDF	130	80
12	Male/45	C_4/5_	CDH	NCR	9	C_4/5_ ACDF	123	100
13	Male/48	C_4/5_ C_5/6_	CDH	NCR and SCC	17	C_5_ ACCF	171	210
14	Male/59	C_4/5_	CDH	NCR	3	C_4/5_ ACDF	108	60
15	Female/39	C_4/5_	CDH	NCR	4	C_4/5_ ACDF	119	100
16	Male/71	C_4/5_ C_5/6_	CDH	ambiguous	8	C_4‐__6_ ACDF	143	120
17	Male/49	C_4/5_	CDH	NCR	6	C_4/5_ ACDF	124	70
18	Female/53	C_4/5_	CDH	NCR	7	C_4/5_ ACDF	108	70
19	Male/50	C_4/5_ C_5/6_	CDH	SCC	5	C_4‐__6_ ACDF	169	130
20	Female/42	C_4/5_ C_5/6_	CDH	ambiguous	7	C_4‐__6_ ACDF	170	150
21	Male/43	C_4/5_	CDH	NCR	4	C_4/5_ ACDF	115	80

NRC, nerve root compression; SCC, spinal cord compression.

### *Radiographic Outcomes*

For all patients, compression at the ventral nerve root or the spinal cord was found in 13 and four patients, respectively. Two patients were found to have compression at both sites, and the compression sites is difficult to be distinguished in two patients. During the postoperative follow‐up, no significant complications occurred. Reviewing the cervical X‐ray and lateral three‐dimensional reconstruction of CT showed that all surgical segments achieved bony fusion.

### *Functional Improvement Evaluated by Clinical Indexes*

#### *Muscle Strength and MMT Score*

At the final follow‐up, the deltoid muscle strength of all participants was 4.09 ± 0.55 on average, which was significantly higher than the preoperative level of 2.29 ± 0.78 (*P* < 0.01). The recovery rate was 66.49% ± 10.04%. The average brachii muscle strength (4.21 ± 0.62) was significantly improved at the final follow‐up compared with the preoperative 3.16 ± 0.94. The recovery rate was 57.12% ± 12.37% (Table 2). Improvement of the most atrophic muscle by manual muscle testing (MMT) revealed that 12 proximal‐type patients were graded excellent, six were good, and three were fair. The improvement rate was 85.7%.

**Table 2:** Statistical analysis of degree of improvement between before operation and last follow‐up

Assessment items		Preoperative	Last follow‐up	*P*	Improvement rate (%)
Muscle strength rating	**Deltoid muscle**	2.29 ± 0.78	4.09 ± 0.55	<0.01	66.49 ± 10.04
	**Biceps brachii**	3.16 ± 0.94	4.21 ± 0.62	<0.05	57.12 ± 12.37
NDI score		38.25 ± 6.10	12.50 ± 2.42	<0.05	
JOA score		6.02 ± 2.11	12.91 ± 3.82	<0.01	62.23 ± 9.23

#### *NDI*

NDI scored an average of 12.50 ± 2.42 at the final follow‐up, which were significantly improved compared with preoperative score of 38.25 ± 6.10 C (Table 2).

#### *C‐JOA*

The mean C‐JOA score improved from 6.02 ± 2.11 preoperatively to 12.91 ± 3.82 postoperatively (*P* < 0.05). The average recovery rate for C‐JOA score at the final follow‐up was 62.23% ± 9.23% (Table 2).

#### *Subgroup Analysis*

Results of NDI, C‐JOA, and deltoid muscle strength showed no significant difference in analysis at 3 months vs 9 months postoperatively. The score of the biceps brachii strength improved from 3.71 ± 0.42 at 3 months to 4.09 ± 0.55 at 9 months (*P* < 0.05) (Table 3).

**Table 3:** Subgroup analysis on the results of functional parameters at different follow‐up time points (NS, no statistically significant difference)

Follow‐up time points	deltoid muscle strength	biceps brachii strength	NDI score	JOA score
3 months	3.94 ± 0.51	3.77 ± 0.42	11.40 ± 2.30	12.71 ± 3.92
9 months	4.09 ± 0.55	4.21 ± 0.62	12.50 ± 2.42	12.91 ± 3.82
*P*	NS	<0.05	NS	NS

## Discussion

Although CSA has been documented for 60 years, the underlying pathogenesis to proximal‐type CSA is still unclear. Some researchers proposed that the impingements were mainly concentrated in ventral nerve root^2^; alternatively, others attribute it to impingement against the anterior horn^6^, ^18^. Studies by Fujiwara *et al*. and Shinomiya *et al*. proposed that the impingement against both the ventral nerve root and the anterior horn might cause disease^10^, ^19^. For the CSA patients in our study, although impingement against both sites were found though MRI examination, the distinction between them was ambiguous especially for those that involve multilevel spinal stenosis.

There is still much controversy around surgical options for patients with CSA. Previously, there were claims that posterior laminoplasty with or without foraminotomy have comparable results with anterior decompression and fusion. A recent study by Chen *et al*. recommended anterior decompression as first choice regardless of the number of spinal canal stenosis considering pathogenic lesion leading to CSA comes from the ventral side of the nerve root or anterior horn^20^. All the patients in our study adopted an anterior approach in the decompression procedure, which achieved satisfactory results in the proximal‐type CSA patients. Nevertheless, potentials of functional improvements in CSA patients after decompression are limited, and most clinical parameters showed no significant improvements at 9 months postoperatively compared with that at 3 months.

It is worth noting that in the decompression procedure of one case, disc tissue was absent in the C_4/5_ intervertebral space and a rupture was found in the posterior longitudinal ligament. After further intraoperative exploration, multiple broken nucleus pulposus were found at the outlet of C_5_ nerve root. Then a complete decompression was performed to the affected nerve roots. Thus, enlarging the intervertebral foramen is needed in such cases to achieve a complete decompression of the affected nerve roots.

It is generally accepted that upper limb muscles are innervated by several nerve roots^21^. C_5_ nerve roots mainly innervate the deltoid muscle, as well as part of the biceps muscle. Damage to C_5_ nerve root compression often results in restricted motion of shoulder and/or elbow, which has been reported to occur in C_4/5_ segment disc herniation. However, some scholars proposed that deltoid muscle spasm may also occur in patients with C_3/4_ or C_5/6_ single‐segment CDH^22^, ^23^. Han *et al*. reported a total of 14 patients with deltoid muscle paralysis caused by single‐segment cervical disc herniation and cervical spondylosis of the nerve root ranging from C_3_ to C_6_ levels^24^. Nevertheless, in some cases, the pathogenesis of decreased deltoid or biceps brachii muscle strength in the C_3/4_ or C_5/6_ segment of the CDH cannot be interpreted solely by clinical studies. Such phenomenon was interpreted as dissociation between the levels of spinal stenosis and atrophic muscles. In our study, eight of 21 patients with C_5_ nerve root paralysis were found to be involved with C_4/5_ and C_5/6_ double‐segmental lesions. After a two‐stage decompression, all patients achieved satisfactory results. For these patients, it is advisable to make a comprehensive surgical plan according to involved levels so as to achieve a complete decompression.

Several limitations of our study must be acknowledged. Firstly, the number of cases was small and therefore insufficient for statistically evaluating the surgical outcomes. This was mainly because CSA patients of this type were rare and there was loss of contact with patients in the follow‐up. Secondly, duration of follow‐up was short. The shortest follow‐up of the study was 10 months, with an average of 13.2 months, which is less persuasive compared with the long‐term clinical follow‐up studies reported in previous literature.

### *Conclusions*

In this study, we demonstrated clinical features of proximal‐type CSA with cervical radiculopathy and evaluated the surgical outcomes of anterior approaches. On the basis of our analysis, we concluded that anterior decompression is effective and can achieve satisfying clinical improvement in both clinical syndrome and muscle strength of atrophic muscles in proximal type CSA patients with cervical radiculopathy.
